# Brain SegNet: 3D local refinement network for brain lesion segmentation

**DOI:** 10.1186/s12880-020-0409-2

**Published:** 2020-02-11

**Authors:** Xiaojun Hu, Weijian Luo, Jiliang Hu, Sheng Guo, Weilin Huang, Matthew R. Scott, Roland Wiest, Michael Dahlweid, Mauricio Reyes

**Affiliations:** 1 Malong Technologies, Shenzhen, China; 2 Shenzhen Malong Artificial Intelligence Research Center, Shenzhen, China; 3 Department of Neurosurgery, Second Clinical Medical College of Jinan University (Shenzhen People’s Hospital), Shenzhen, China; 4 Imaging A.I. Lab, Insel Data Science Center, Bern University Hospital, Bern, Switzerland

**Keywords:** Brain tumor segmentation, Stroke outcome prediction, 3D brain MRIs, Curriculum learning

## Abstract

MR images (MRIs) accurate segmentation of brain lesions is important for improving cancer diagnosis, surgical planning, and prediction of outcome. However, manual and accurate segmentation of brain lesions from 3D MRIs is highly expensive, time-consuming, and prone to user biases. We present an efficient yet conceptually simple brain segmentation network (referred as Brain SegNet), which is a 3D residual framework for automatic voxel-wise segmentation of brain lesion. Our model is able to directly predict dense voxel segmentation of brain tumor or ischemic stroke regions in 3D brain MRIs. The proposed 3D segmentation network can run at about 0.5s per MRIs - about 50 times faster than previous approaches Med Image Anal 43: 98–111, 2018, Med Image Anal 36:61–78, 2017. Our model is evaluated on the BRATS 2015 benchmark for brain tumor segmentation, where it obtains state-of-the-art results, by surpassing recently published results reported in Med Image Anal 43: 98–111, 2018, Med Image Anal 36:61–78, 2017. We further applied the proposed Brain SegNet for ischemic stroke lesion outcome prediction, with impressive results achieved on the Ischemic Stroke Lesion Segmentation (ISLES) 2017 database.

## Background

Accurate brain lesion segmentation from MR images (MRIs) is of great importance to improving cancer diagnosis, surgery planning and prediction of patient outcome, such as brain tumor and stroke lesion. However, manual and accurate segmentation of brain lesions from 3D MRIs is highly expensive, time-consuming, and prone to user biases. Efforts have been devoted to developing automatic methods for this task, but it is still challenging to precisely identify brain lesions, which are often diffused (e.g., infiltrative grade III and IV brain tumors), poorly contrasted, and their boundaries are easily confused with healthy brain tissues. For example, structural tumor regions, such as necrotic core, oedema and enhancing core, can appear in any location of the brain with various sizes and shapes, making it particularly difficult to segment them accurately. Particularly, making a rapid clinical decision for stroke lesion has a critical impact to patient treatment outcome. Therefore, automatic prediction of the stroke lesion outcome has a great potential to improve stroke assessment, which would greatly facilitate the physician’s decision making process. To improve the performance, multiple MRI modalities, such as T1, T1-contrast, T2 and Fluid Attenuation Inversion Recovery (FLAIR) for glioblastomas, or diffusion and perfusion MRI (e.g., TTP, MTT, Tmax, rCBF, rCBV) for stroke patients, are often utilized to provide richer visual information, and automatic methods are developed to explore such multiple MRI modalities for learning and prediction.

Past work in the literature has been dominated by approaches that pose brain lesion segmentation as the problem of sematic segmentation, which produces dense pixel-wise classification slice by slice. Recent deep convolutional neural networks (CNNs) have been applied to this task by advancing feature learning and representation. These approaches learn deep, hierarchical features from brain MRIs, allowing the model to learn deep features with a integrated classifier simultaneously. This is different from traditional approaches that often use manually-designed features or CNN features, where a classifier (e.g., support-vector-machine (SVM) is trained separately. For example, Liu *et. al.* combined CNN features and a SVM classifier to automatically predict genotypes from brain MRIs [3]. An end-to-end learning allows features learning and classifier training to work collaboratively, resulting in a more meaningful and stronger deep representation that leads to state-of-the-art performance on brain tumor segmentation [1, 2, 4–7] and stroke lesion segmentation [8–12].

However, recent CNN approaches for brain lesion segmentation often suffer from several common limitations that negatively impact their performance. First, CNNs are powerful to compute high-level context features with multi-layer hierarchical designs where multiple pooling operations are applied. It is commonly accepted that a CNN model has stronger capability with a deeper design, by increasing the number of convolutional layers [13, 14]. However, a CNN with multiple pooling operations gives raise to a significant issue of down-scaling of convolutional feature maps, leading to information loss on fine structures and detailed context through the pooling layers. Such local detailed information is critical to accurate segmentation of lesion regions from brain MRIs. Second, segmentation often involves dense training and inference where training samples are highly correlated with their neighboring pixels, and thus data unbalance among various classes and background often happens, and is becoming a significant issue. This makes it difficult to train a high-performance 3D segmentation model. Third, it is challenging to effectively aggregate meaningful 4D information over multiple MRI modalities by using a single CNN model.

In this work, we present a new 3D brain segmentation network built on a deep residual architecture [14]. The approach we describe in this paper is a single-model 3D segmentation CNN that can work efficiently for brain tumor segmentation and ischemic stroke lesion outcome prediction. Our model is able to directly predict dense voxel-level segmentation results of various tumor tissues or ischemic stroke lesion from 3D brain MRIs, *without any post-processing*. Our method is related to that of [2] which inspired the current work, where a 3D convolutional architecture combines multi-scale MRIs with a Conditional Random Field (CRF) [15] for post-processing. The current paper describes a method that integrates a number of key technical developments into a single 3D CNN model. Our contributions are described as follows:

- We propose a new 3D refinement module capable of aggregating rich fine-scale 3D deep features from multi-modality brain MRIs over multiple 3D convolutional layers. This allows us to extract both local detailed features and high-level context information in 3D domain, which is critically important to achieving accurate segmentation at the voxel level.
- We introduce a new training strategy that incorporates curriculum learning and a recently proposed Focal loss into our 3D segmentation networks. This allows our model to learn more efficiently with the designed curriculum, where the issue of dense training and class imbalance are handled effectively and efficiently.
- We integrate these technical improvements into a single model which allows for a direct prediction of dense voxel-level segmentation in *one pass*. This results in a highly efficient model running at about 0.5s per MRIs - allowing for deployment of the solution in clinical scenarios where computation time is critical, as it is the particular case of stroke. We report state-of-the-art results on the BRATS 2015 [16] and the ISLES 2017 [17], for brain tumor segmentation and stroke lesion outcome prediction, respectively.

## Methods

In this section, we describe details of the proposed Brain SegNet, which is a 3D refinement network including a designed 3D refinement module, and a new training strategy that integrates curriculum learning [18, 19], Focal loss [20] and data augmentation.

### Overview

**Fig. 1:**
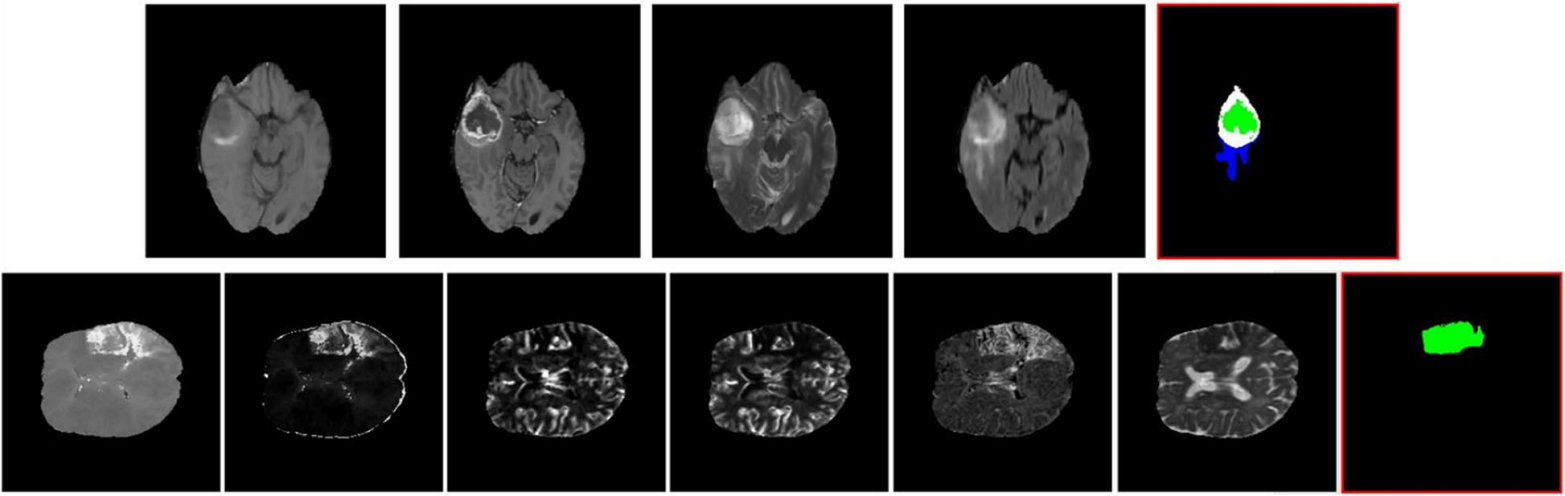
Examples of multi-modality brain MIRs for brain tumor segmentation (from BRATS 2015 database [16]) and stoke lesion segmentation (from ISLES 2017 database [17]). Top (left → right): MRI modalities of {T1, T1-contrast, T2, FLAIR} and brain tumor segmentation results (with a RED bounding box); Bottom (left → right): MRI modalities of {TTP, Tmax, rCBV, rCBF, MTT, ADC} and stoke lesion outcome prediction results

Our goal is to precisely estimate the label of tumor tissues or stroke lesions at each voxel, by using multiple 3D MRI modalities as input, such as {T1, T1-contrast, T2, FLAIR} MRIs for brain tumor segmentation, or {TTP, Tmax, rCBV, rCBF, MTT, ADC} MRIs for stroke outcome prediction, as shown in Fig. 1. For brain tumor segmentation, our model predicts four tumor tissue sub-compartments - necrotic core, oedema, non-enhancing and enhancing core, as defined in [16]. An exemplar image is shown in Fig. 1, where different colors in the predicted results indicate various tumor tissues. Stroke lesion outcome prediction is a binary segmentation task, as shown in Fig. 1, describing expected brain tissue loss post-mechanical thrombectomy on follow-up imaging.

**Fig. 2:**
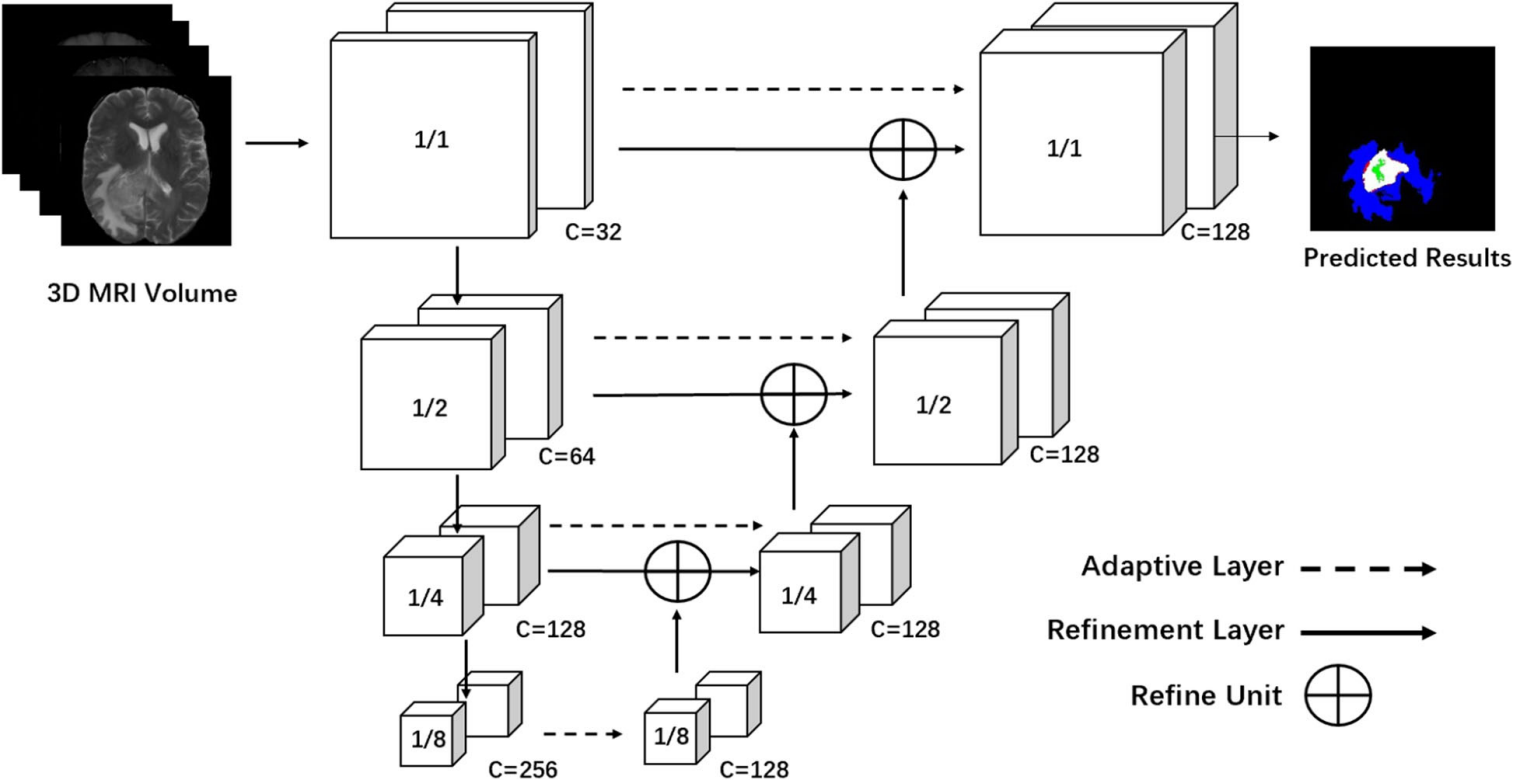
Architecture of the proposed 3D brain segmentation network (Brain SegNet) for brain lesion segmentation from MRIs. The input is multi-modality 3D MRI volume data. It has four convolutional blocks, and contains 17 convolutional layers in total, with residual units. It includes a refinement module capable of aggregating rich fine-scale 3D volume features over multiple convolutional blocks. An adaptive layer and an refinement layer are applied to each block for computing multi-level convolutional features

In this work, we cast ResNet [14], originally designed for image classification, into 3D dense segmentation from brain MRIs. Essentially, a 2D semantic segmentation task can be considered as a dense classification problem implemented on each image patch corresponding to a dense pixel at the output layer. Specifically, we make two major modifications on the original ResNet consisting of four convolutional blocks by using 3×3 convolutional kernels. First, we introduce 3D convolutions with a kernel size of 3×3×3 in all convolutional layers, as shown in Fig. 2. The 3D convolutional filters naturally take all multiple MRI modalities as input, by considering each modality as a 3D channel. This allows for an arbitrary number of MRI modalities as input. Second, the output prediction maps should have the exactly same spatial resolution as that of the input MRIs, to ensure a dense prediction. The output layer is built on the last convolutional layer by computing a multi-class soft-max function at each spatial location, e.g., 5 classes for brain tumor segmentation and 2-class segmentation task for stroke lesion outcome prediction, both of which include a background class. Therefore, the spatial resolution of the last convolutional layer should be amplified and aligned to that of an input volume. This is achieved by employing the proposed 3D refinement module which is described in “Results on Stroke Lesion Outcome Prediction” section.

**Fig. 3:**
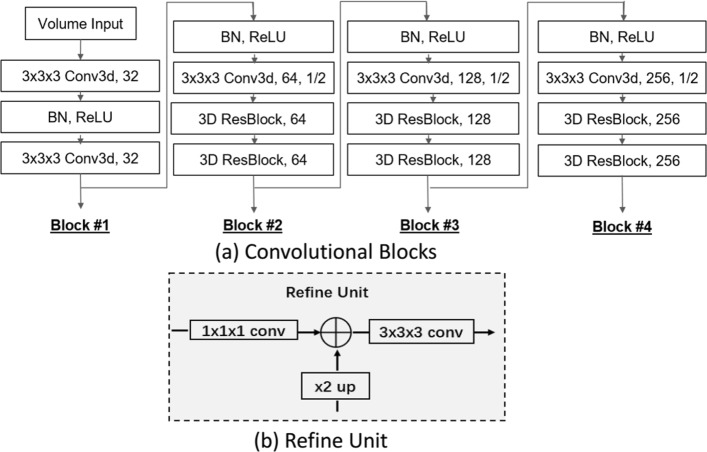
**a** Layers of the proposed 3D model in four convolutional blocks. **b** Details of the proposed refine unit

Our 3D segmentation model consists of four 3D convolutional blocks, each of which contains a number of layers by using 3D convolution, with Rectified Linear Unit (ReLU) [21] and Batch Normalization (BN) [22] operations which are applied empirically by following [14]. Details of the four blocks are presented in Fig. 3a. Specifically, all convolutional feature maps within each convolution block have an identical 4D shape (a same number of channels with an identical 3D shape for all channels). The number of channels is increased from Block#1 to Block#4 as {32, 64, 128, 256}. In each block, a 3×3×3 convolution is used to down-sample the 3D maps to half in all dimensions, resulting in down-sample factors of $\left \{1, \frac {1}{2}, \frac {1}{4}, \frac {1}{8}\right \}$ in four blocks. We explore convolution operations for down-sampling the convolutional maps, which are different from the original ResNet [14] using max pooling operations for extracting high-level semantic context features for image classification. The convolution operations capable of preserving richer local details, which is important to dense segmentation task. Our model contains two ResBlocks in the Block #2, #3, #4, and each ResBlock has two convolutional layers with residual connections designed by following ResNet [14]. This results in 17 3D convolutional layers in total for our segmentation model, which is a compact design, compared to the original ResNet [14], resulting in fast running speeds. At the same time, this also allows our model for requiring a smaller amount of training data, while attaining better levels of generalization than models requiring more parameters, and hence, larger training datasets.

### 3D Refinement Module

For dense 3D segmentation, a key step is to align the spatial shape of convolutional maps to the input volume. A straightforward approach is to use up-sample operations. However, the up-sample operation commonly suffers from a significant issue of down-scaling of the feature maps, leading to information loss of fine structures and local details [23]. Both fine structures and high-level context information are important to many segmentation tasks. To effectively encode both of them in the convolutional features, we propose a novel 3D refinement module, with the goal of aggregating local details and multi-level high-level context information in 3D volume domain.

As shown in Fig. 2, the proposed 3D refinement module is applied to each convolutional block. It recursively encodes the high-level context information from the higher layers to the lower ones in 3D volume domain. Our 3D refinement model works as follows. First, we design an adaptive layer which is connected to the output of each convolutional block. The adaptive layer reshapes the 4D convolutional maps by changing the number of 3D channels to a constant number - in our experiment, 128, while keeping the 3D shape of convolutional features in each channel. Second, a refine unit is designed and applied to fuse multi-level convolutional features recursively from the top convolutional block to the bottom one. As shown in Fig. 3b, we introduce 1×1×1 and 3×3×3 convolutional layers to refine the 3D convolutional features before and after the combination operation, where an element-wise summation is applied, with an ×2 up-sample operation implemented on the lower-resolution maps. This makes it different from the skip connection introduced in [23] where direct element-wise summation was applied.

This results in 128-channel 3D convolutional maps in the last refined convolutional layer, and each 3D convolutional maps have the same shape with the input 3D volume. The final multi-class voxel-wise prediction is computed on the 128-dimensional features at each voxel location of the 3D feature maps. In our model, we adopt 1×1×1 kernels for all adaptive layers for simplicity, but more advanced configurations, such as a non-local operation, a dilated convolution, or an inception architecture, is readily applicable, and may have the potential to improve the performance. Our goal here is to present a baseline framework for the 3D refinement module.

Finally, the 3D refinement module generates high-resolution 3D semantic maps, which enrich feature representation in the convolutional layers. It can be integrated seamlessly into our 3D segmentation network, resulting in an end-to-end trainable network that improves the performance.

### Training 3D Segmentation Network with Curriculum

An efficient training approach is critically important to exploring the full potential of our 3D segmentation model, particularly by using a very limited number of brain MRIs for training. We present a new training strategy for end-to-end learning of 3D brain segmentation network, which incorporates with curriculum learning [18], Focal loss [20] and data augmentation. We emphasize that high performance of our 3D model not only depends on its architecture design, but also due to the new training strategy proposed.

As mentioned, a segmentation task can be considered as a dense classification problem, where a training loss is computed *densely* over all 3D volume locations of a MRI scanning. This rises a number of significant issues during training. First, dense 3D training often generates a large number of training samples, which are significantly redundant when the model learns from neighboring samples within a 3D volume. These samples are closely relevant with small diversity, and thus are less informative. Second, training is inefficient when dominated by a large amount of “easy" samples, which often contribute to the learning process with less useful signal or information. This happens in dense 2D image detection [20], and becomes more significant in dense 3D segmentation task. Our training approach is developed to cope with these critical issues, and is explained below.

#### Focal Loss

As stated in [20], using automatically-selected meaningful samples is critical to learn a high-capability model for a dense estimation task. Focal loss was originally introduced in [20] for general object detection. It encourages the model to learn from a sparse set of hard samples, which naturally alleviates negative impact from the vast amount of easy samples, leading to performance boost. Formally, Focal loss was defined by introducing a modulating factor (*γ*) to a cross entropy loss [20]:

1 $$ FL(p_{t}) = -(1-p_{t})^{\gamma}\log(p_{t})  $$

where *p*_*t*_=*p* if *y*=1, and otherwise *p*_*t*_=1−*p*. *y*∈{−1,+1} is the ground-truth class, and *p*∈[0,1] is the estimated probability for the class of *y*=1. *γ* is a focusing parameter. Focal loss is equal to the original cross entropy loss when *γ*=0, and the training would focus on hard samples when *γ*>0. It down-weights “easy" samples smoothly. An “easy" sample has a large value of *p*_*t*_, indicating a high estimated probability for a correct class. A larger value of *γ* means stronger contribution from a hard sample to the training process. In this work, we cast Focal loss into our 3D segmentation framework by replacing the original soft-max loss, providing a simple formulation that allows the model to automatically select a spare set of meaningful samples for learning.

#### Data Augmentation

The input of our model is 3D volumetric MRIs, and the number of training samples is often limited. Data augmentation provides a straightforward approach for increasing the amount of training samples, allowing us to generate massive scale training data with increasing diversity. In our experiments, data augmentation is produced as follows. First, a simple slice-level augmentation is implemented by randomly amplifying voxel intensities with an amplification factor selected randomly from 0.8-1.2. Second, we produce 3D volume-level augmentation where exact same operations are implemented through all slices within a 3D volume: (i) all slices are rotated with a same orientation, which is randomly selected from {0, 90, 180, 270} degrees; (ii) all slices are re-scaled by using a same ratio randomly computed from [0:5; 1:2]; (iii) horizontal and vertical flippings are implemented sequentially; (iv) random cropping: a same spatial cropping is further implemented on each slice, and each cropped region should include a whole region of interest (e.g., brain tumor or stroke region), if it is presented in the slice. Data augmentation and Focal loss are incorporated and form a learning curriculum for our new training strategy.

#### Learning with Curriculum

Both Focal loss and data augmentation encourage the model to learn from data with larger diversity and more complexity. However, as shown in our experiments, directly applying these technologies to our 3D segmentation model does not directly yield obvious performance gains, and hence an efficient learning scheme is critical. Our training scheme is inspired from the intuition of curriculum learning [18], which encourages the learning to start from an easier task, and then takes more complex tasks gradually during the training process. We propose a three-stage learning curriculum where data complexity is increased gradually. This allows for an efficient implementation of curriculum learning specifically designed for our task of brain segmentation. Full process is described as follows.

- First, our 3D segmentation model is trained on the original data without data augmentation and Focal loss. This can be considered as an easy task, and the model often converges fast, due to the small amount of training data which is often dominated by easy samples.
- Then we increase data complexity by randomly applying data augmentation (described above) to 50% of the original 3D MRIs, which generates training samples with large diversity. We continue the training process by using the generated data, which naturally increases the difficulty of the learning task.
- Finally, in the last stage of the proposed learning curriculum, we apply Focal loss which encourages the model to learn from harder samples. In addition, we further increase data complexity by implementing data augmentation to 75% of training data. Both operations further increase the learning difficulty by using more meaningful and discriminative samples, which are of great importance to improving model capability.

The proposed three-stage curriculum allows our models to learn more efficiently from a limited amount of training samples, by increasing data complexity gradually. This leads to stronger discriminative power of the trained models with better generalization capability, resulting in clear performance improvements.

### Discussions

Our Brain SegNet is related to 3D U-Net [24] or V-Net [25], but has clear distinctions. It is designed for brain lesion segmentation from multi-modality MRIs, where fine detailed information in 3D volume domain is of great importance, while 3D U-Net or V-Net was developed for a different task, leading to the major difference in model design. (1) Our 3D segmentation network is more compact in design, with less convolutional layers in the decoding branch. This results in a large reduction in model parameters. For example, the parameters of our model is 12M, compared to 19M of the 3D U-Net, and to the 60M V-Net. With less parameters, our model can be trained more efficiently by using a decreasing number of training samples. Furthermore, this allows our model to run significantly faster - at about 0.5s per MRI, which is over x50 faster than related approaches described in [2] and [1], both of which are state-of-the-art approaches for this task. (2) Our 3D model only implements down-sample pooling in spatial domain, and keep its third dimension unchanged in all convolutional layers. This allows it to preserve strong 3D detailed information, tailored it towards brain lesion segmentation, where 3D volume information from multiple modalities is critical to distinguish various brain tissues in fine details. Furthermore, our 3D segmentation network is trained with a new proposed training strategy, and the whole design pipeline is unique and efficient, leading to new state-of-the-art performance.

Our work is also related to that of [2, 6], where 3D CNNs were developed for voxel-level segmentation. In [6], Chen *et. al.* developed a voxel-wise residual network (VoxResNet) for segmentation of key brain tissues. VoxResNet extends deep residual architecture [14] by replacing 2D convolutions with 3D convolutions, and integrates the features learned from multiple MRI modalities. Kamnitsas *et. al.* [2] proposed a dual pathway 3D CNN for aggregating multi-level features, and a CRF was applied to refine the results. We develop a 3D refinement module, setting it apart from that of [2, 6]. Importantly, our segmentation model learns multi-modality information and aggregates multi-level 3D convolutional features with a single CNN model that produces inference in one pass. This provides a more compact yet accurate model that works more efficiently and faster.

### Implementation Details

The proposed 3D Brain SegNet was implemented in Pytorch. In the training stage, we generate a number of fixed-size 3D volumes (which are used as a channel for 4D inputs) from each 3D MRIs modality. For brain tumor segmentation on the BRATS database [16], each MIR scanning has a fixed-number of 155 slides, and we set the size of each 3D volume to 12x128x128, where 12 is the number of slides, and 128x128 is the spatial resolution. Each MRI sequence is sampled equally with non-overlap in the third dimension, and the spatial region is obtained by using random cropping, which is similar to the random cropping method described in data augmentation. Therefore, each 155-slide MRI scanning from the BRATS database generates 13 cropped 3D volumes as model inputs. For stroke lesion outcome prediction from the ISLES 2017 database [17], the size of 3D volume is set to 7x96x96, and the number of slides for each MRI scanning is about 20, which generates 3 cropped 3D volumes.

Our 3D models were trained on 4 Titan Xp GPUs with 1500 epochs, by using a batch size of 40, which takes about 8G memory for each GPU used. We adopted Adam optimization, and set a learning rate to 1e-4. For the proposed three-stage training strategy, we simply trained a model with 500 epochs in each learning stage, while keeping other settings fixed. Therefore, training time is varied over different databases or tasks, due to various scales of the databases. For example, the training takes about eight hours on the BRATS 2015 database, while the training on the ISLES 2017 database just uses about one hour, due to the small number of training samples.

In the inference stage, we split each brain MRI scanning into five sub-volumes with a size of 31x240x240 in order to produce inference with a single GPU, due to the limitation in GPU memory. We predict each sub-volume individually, and concatenate the outputs of the five sub-volumes to form the final results. Each MRI scanning takes about 500ms in inference, including the time of data preparation. The final predicted labels are obtained by taking the maximum probability of each class, without any post-processing. Notice that without GPU memory limitation, our model allows for an input of arbitrary size in inference. For example, on the ISLES database, our model can predict a whole MRI scanning in a single pass, due to the fewer number of slices contained in the MRI scannings from this database, as compared to the BRATS set.

## Results

The proposed 3D Brain SegNet is evaluated on two brain lesion segmentation tasks: brain tumor segmentation and ischemic stroke lesion outcome prediction (which is also a segmentation task). We use BRATS 2015 [16] database for brain tumor segmentation, which is a widely-used benchmark for this task. ISLES 2017 database [17] is used for ischemic stroke lesion outcome prediction.

### Results on Brain Tumor Segmentation

**Database.** BRATS [16] database has 220 cases with high grade (HG) and 54 cases with low grade (LG) glioma in training set, with segmentation ground truth (GT) provided. The task is to segment four tumor tissues: necrotic core, oedema, non-enhancing and enhancing core. The test set contains 110 cases. We follow [2] by merging the four predicted labels into different sets: a *whole* tumor for combining four classes, a *core* tumor for class 1,3,4, and an *enhancing* tumor for just class 4. FLAIR, T1, T1-contrast and T2 modalities are available. Results are measured by using standard *Dice* score, *sensitivity* (true positive rate) and *specificity* (true negative rate), which are computed as follows,

2 $$ Dice = \frac{|(P_{=1})\bigcap(T_{=1})|}{(|P_{=1}|+|T_{=1}|)/2} \quad  $$

3 $$ Sens. = \frac{|(P_{=1})\bigcap(T_{=1})|}{|T_{=1}|} \quad  $$

4 $$ Spec. = \frac{|(P_{=0})\bigcap(T_{=0})|}{|T_{=0}|}  $$

where $\bigcap $ is the logical AND operator, |·| is the number of voxels belonging to the set. *P*∈{0,1} and *T*∈{0,1} are model prediction and ground truth respectively. *P*_=1_ and *T*_=1_ represent the set of voxels where *P*=1 and *T*=1. The *Dice* score normalizes the number of true positives to the average size of the two segmented areas, and is identical to the *F-measure*.

**Fig. 4:**
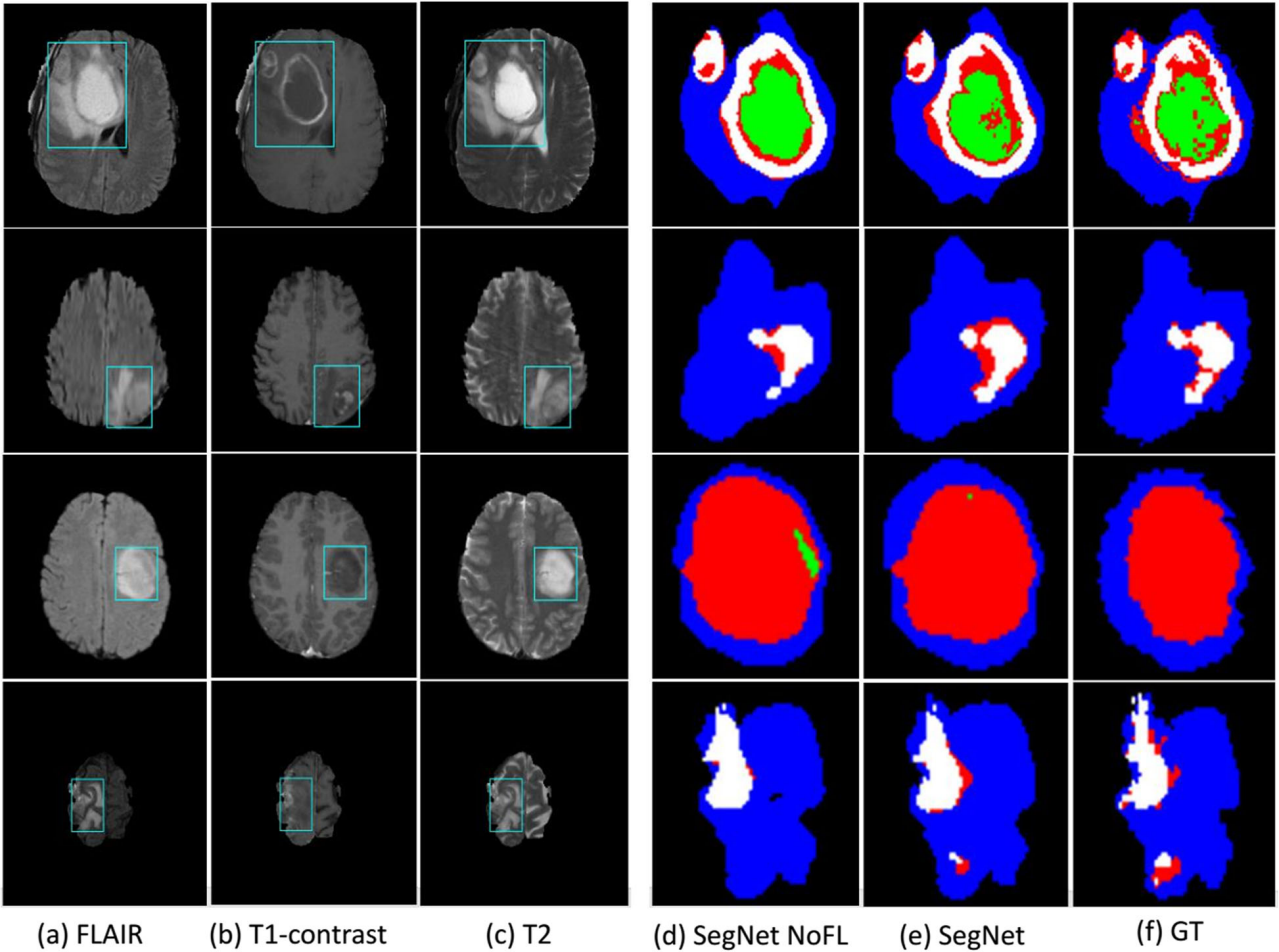
Segmentation results on several examples. **a** FLAIR, **b** T1-contrast, **c** T2, **d** results of Brain SegNet without Focal loss, **e** results of Brain SegNet with Focal loss, and **f** ground truth. Colors: necrotic core (red), oedema (green), non-enhancing core (blue), and enhancing core (white)

**Experimental results.** Our model is evaluated on the full test set of the BRATS, in the terms of standard *dice* score, *specificity* and *sensitivity*, which are computed by using the online evaluation platform provided by the organizers. The results and comparisons are presented in Table 1, and the predicted results on a number of examples are demonstrated in Fig. 4. As shown in Table 1, our 3D segmentation network trained by using the designed curriculum with a Focal loss achieved the best performance on the *whole* tumor and the *enhancing* tumor in the term of *dice* score, which is the most important measurement that balances *specificity* and *sensitivity*. Our model with the new training strategy leads to clear performance improvements, particularly for the *core* tumor. Furthermore, Focal loss can improve the performance of the *enhancing* tumor with a *dice* score of 0.69 →0.72, and the *whole* tumor with a *dice* score of 0.85 → 0.86. Focal loss encourages the model to learn from hard samples, which enable it with stronger discriminative capability for identifying more fine-grained details on the boundary of tumors, as demonstrated in Fig. 4.

**Table 1:** Evaluation on the test set of the BRATS 2015 (110 testing cases), with comparisons with the most recent results reported in [1, 2]

	Dice			Specificity			Sensitivity		
	Whole	Core	Enh.	Whole	Core	Enh.	Whole	Core	Enh.
Zhao et. al.[1] + CRF	82.0	72.0	62.0	84.0	78.0	60.0	83.0	73.0	69.0
Zhao et. al.+3D CRF [1]	84.0	73.0	62.0	89.0	76.0	63.0	82.0	76.0	67.0
DeepMedic [2]	83.6	67.4	62.9	82.3	84.6	64.0	88.5	61.6	65.6
DeepMedic+CRF [2]	84.7	67.0	62.9	85.0	84.8	63.4	87.6	60.7	66.2
Brain SegNet_no_FL	85.0	69.0	64.0	88.0	86.0	63.0	85.0	63.0	69.0
Brain SegNet	86.0	72.0	64.0	87.0	85.0	64.0	87.0	68.0	66.0

By comparing with recent state-of-the-art results reported in [1, 2], our model obtains an improvement of about 2%, which is significant for this challenging task. Furthermore, as reported in [1] and [2], both methods employed a CRF for post-processing, in an effort to improve the performance, resulting in about 1-2% performance gains. Our model works more efficiently by running single-stage segmentation, which predicts all results in one pass, without any post-processing step. Thus our method is a more efficient approach that can run at about 0.5s per MRIs scanning (on the BRATS dataset) using a single GPU, which can be applied to real-world applications. This is about ×50 and ×240 faster than that of [2] (taking about 30s) and [1] (using 2-4 mins).

### Results on Stroke Lesion Outcome Prediction

**Database.** ISLES 2017 database [17] contains a total of 75 cases, which were divided into a set of 43 cases for training, and a set of 32 cases for testing. Each case has multiple MRI modalities, containing a raw 4D PWI, five 3D MRI perfusion maps (rCBF, rCBV, MTT, TTP, Tmax), and one 3D MRI diffusion map (ADC). The segmentation ground truth for all cases in the training set were provided by a clinician on a 90-day follow-up. All MRI maps are co-registered and skull-stripped, by following [26].

**Fig. 5:**
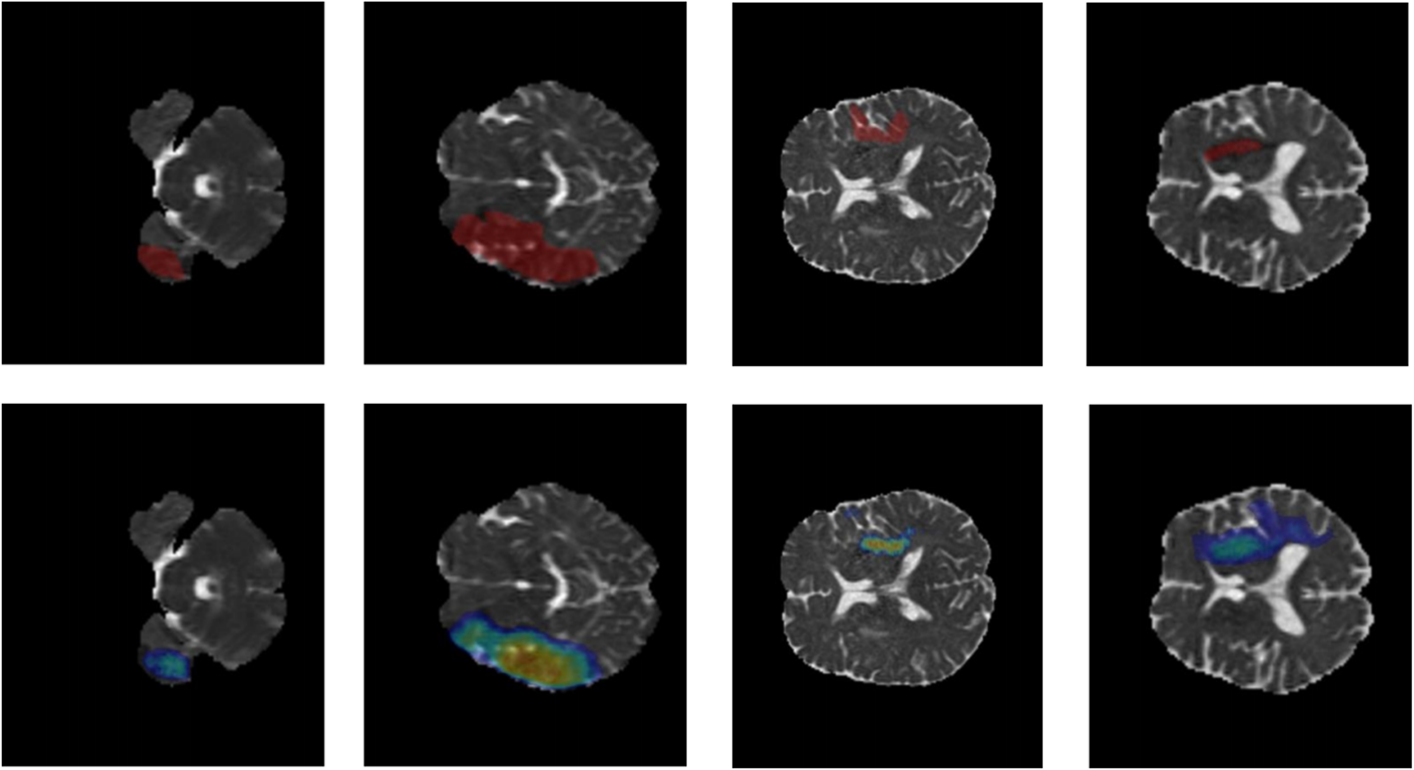
Segmentation results of ischemic stroke lesion on a number of MRI slides (3D MRI diffusion map (ADC)). (Top): clinician results; (Bottom): model prediction (color indicates the predicted probability at each pixel.)

**Experimental results.** The proposed Brain SegNet is further evaluated on the test set of ISLES 2017, in the terms of *dice* score, *precision* and *recall*, by following the online evaluation platform [17] and [8]. The results are reported in Table 2, with the predicted results on a number of exemplar slices which are demonstrated in Fig. 5. As shown in Fig. 5, the proposed model can roughly predict the correct regions of ischemic stroke lesion as clinician expert performed on the presented MRI slides. As shown in Table 2, by using the proposed training strategy, Focal loss improved our *recall* significantly, leading to a clear performance improvement on *dice score*: from 0.26 to 0.30.

**Table 2:** Evaluation results on the test set of ISLES 2017 (containing 32 cases), with comparisons with recent results reported in [8]

	*Dice*	*Precision*	*Recall*
Standard Model [8]	0.20 ±0.19	0.16 ±0.20	0.61 ±0.28
4D-PWI Model [8]	0.20 ±0.18	0.18 ±0.21	0.61 ±0.27
Multi-Data Model [8]	0.26 ±0.21	0.21 ±0.20	0.61 ±0.28
Multi-Data Multi-Model [8]	0.29 ±0.21	0.23 ±0.21	0.66 ±0.29
Brain SegNet_no_FL	0.26 ±0.22	0.35 ±0.28	0.38 ±0.29
Brain SegNet	0.30 ± 0.22	0.35 ±0.27	0.43 ±0.27

Furthermore, we compare our results against most recent results reported in [8], where the authors trained two models separately: one from standard diffusion and perfusion MRIs (e.g., Tmax, TTP, MTT, rCBF, rCBV), and the other from 4D PWI. Then two models were combined in an effort to improve the performance, demonstrating that the 4D PWI can provide complementary information for the standard diffusion and perfusion MRIs for ischemic stroke lesion outcome prediction. As shown in Table 2, a single model trained on both 4D PWI and standard MRIs can improve the *dice score* from 0.20 to 0.26, and combining two models further improved the *dice score* to 0.29. Our single model can obtain a *dice score* of 0.30, which is compared favourably against the best results of [8] achieved by combing two models. Our model obtained a significant improvement of *precision*. Importantly, our model can run at about 0.1s per MRI from ISLES 2017 database, which is significantly faster than that of [8] which takes about 30s per MRI. These results strongly demonstrate the efficiency and effectiveness of our methods for the task of ischemic stroke segmentation from brain MRIs.

## Conclusions

We have presented a new 3D brain segmentation network (Brain SegNet) for automatic segmentation of brain lesion from 3D MRIs, such as brain tumor and ischemic stroke lesion. We proposed a novel 3D refinement module that directly aggregates both local details and 3D semantic context information within 3D convolutional layers. Furthermore, we introduced a new training strategy that incorporates curriculum learning and Focal loss, allowing us to improve model generalization capability by increasing data complexity gradually. These technical improvements are integrated elegantly into a single 3D segmentation CNN, which is a highly-compacted and an end-to-end trainable model that can run at about 0.5s or 0.1s per MRI volume, which is significantly faster than previous approaches. The proposed methods obtained the state-of-the-art performance on both BRATS 2015 database for brain tumor segmentation, and ISLES 2017 database for ischemic stroke lesion outcome prediction.

## Data Availability

The datasets analysed during this current study are available in the BRATS 2015 and ISLES 2017.

## Abbreviations

BN: Batch normalization
CNNs: Convolutional neural networks
CRF: Conditional random field
FLAIR: Fluid attention inversion recovery
GT: Ground truth
HG: High grade
ISLES: Ischemic stroke lesion segmentation
MRIs: Magnetic resonance images
ReLU: Rectified linear unit
